# Ethnic differences in prostate-specific antigen levels in men without prostate cancer: a systematic review

**DOI:** 10.1038/s41391-022-00613-7

**Published:** 2022-12-01

**Authors:** Melissa Barlow, Liz Down, Luke Timothy Allan Mounce, Samuel William David Merriel, Jessica Watson, Tanimola Martins, Sarah Elizabeth Rose Bailey

**Affiliations:** 1 University of Exeter, St Luke’s Campus, Heavitree Road, Exeter, Devon EX1 2LU UK; 2 University of Bristol, Bristol Medical School, Bristol, BS8 1TH UK

**Keywords:** Diagnostic markers, Prostate cancer

## Abstract

**Introduction:**

Black men are twice as likely to be diagnosed with prostate cancer than White men. Raised prostate-specific antigen (PSA) levels can indicate an increased risk of prostate cancer, however it is not known whether PSA levels differ for men of different ethnic groups.

**Methods:**

*PubMed* and *Embase* were searched to identify studies that reported levels of PSA for men of at least two ethnic groups without a prostate cancer diagnosis or symptoms suggestive of prostate cancer. An adaptation of the Newcastle-Ottawa scale was used to assess risk of bias and study quality. Findings were stratified into the following broad ethnic groups: White, Black, Asian, Hispanic, and Other. Data were analysed in a narrative synthesis due to the heterogeneity of reported PSA measures and methods in the included studies.

**Results:**

A total of 654 197 males from 13 studies were included. By ethnicity, this included 536 201 White (82%), 38 287 Black (6%), 38 232 Asian (6%), 18 029 Pacific Island (3%), 13 614 Maori (2%), 8 885 Hispanic (1%), and 949 Other (<1%) men aged ≥40 years old. Black men had higher PSA levels than White men, and Hispanic men had similar levels to White men and lower levels than Black men.

**Conclusions:**

Black men without prostate cancer have higher PSA levels than White or Hispanic men, which reflects the higher rates of prostate cancer diagnosis in Black men. Despite that, the diagnostic accuracy of PSA for prostate cancer for men of different ethnic groups is unknown, and current guidance for PSA test interpretation does not account for ethnicity. Future research needs to determine whether Black men are diagnosed with similar rates of clinically significant prostate cancer to White men, or whether raised PSA levels are contributing to overdiagnosis of prostate cancer in Black men.

## Introduction

Prostate cancer is the second most common cancer and the fifth leading cause of cancer death in males worldwide with 357 000 annual deaths [1]. Incidence and mortality of prostate cancer differ according to ethnicity: each are twice as high in Black males compared to White males in both the USA [2, 3] and the UK [4–6], whereas Asian men in the UK experience lower rates [4–6].

Prostate-specific antigen (PSA) is a protein secreted by the prostate gland and is measured through blood testing. PSA can be elevated in patients with prostate cancer or benign prostate disease; it does not accurately discriminate between the two and the benefits of PSA screening are unclear [7–11]. Evidence on the diagnostic accuracy of PSA in symptomatic men is focussed on the referred population, and the performance in men consulting primary care is unknown [12]. The most recent systematic review of the diagnostic accuracy of PSA for prostate cancer in patients with lower urinary tract symptoms found that a PSA threshold of 4 ng/mL had a sensitivity of 0.93 (95% CI 0.88, 0.96) specificity of 0.20 (95% CI 0.12, 0.33), and the Area Under the Curve (AUC) was 0.72 (95% CI 0.68, 0.76), although this did not factor in patient ethnicity and studies were at high risk of bias [13].

To assess a patient’s risk of prostate cancer in the USA, doctors are encouraged to use their clinical judgement of a patient’s PSA level in combination with factors that elevate their risk (such as whether a patient is of Black ethnicity). Patients are then referred for further tests or monitoring if necessary [14]. In the UK, guidance from the National Institute of Health and Clinical Excellence (NICE) provides age-specific PSA thresholds to assess a patient’s risk of prostate cancer [15]. These guidelines recommend that men with a PSA above age-specific thresholds should be offered investigation and referral for suspected prostate cancer, but does not take into account the patient’s ethnicity. A recent systematic review found ethnicity to be a considerable source of heterogeneity when assessing age-adjusted PSA reference ranges and concluded ethnicity should be considered when clinically assessing PSA levels [16].

There is currently no ethnicity specific guidance for interpreting PSA results although previous studies have considered ethnicity-specific PSA thresholds [17–19]. Identifying differences in PSA levels in men of different ethnic groups without prostate cancer could help refine the identification of men who may benefit from investigation for suspected prostate cancer. This systematic review sought to identify studies that reported PSA levels for different ethnic groups for men without a prostate cancer diagnosis or symptoms suggestive of prostate cancer and incorporated the findings from these studies into a narrative synthesis to determine the effects of ethnicity on PSA.

## Methods

### Protocol

This systematic review closely adhered to the study protocol which was published on the PROSPERO website on the 29^th^ September 2021 before commencement of abstract screening (reference CRD42021274580) and was conducted in strict accordance to the PRISMA 2020 reporting guidelines.

### Eligibility criteria

This review aimed to identify studies that reported measures of PSA levels of men without a prostate cancer diagnosis or symptoms suggestive of prostate cancer for at least two different ethnic groups. Studies that only included PSA values for only one ethnic group were excluded to reduce selection, design, measurement, and reporting bias. We included observational studies and randomised controlled trials with baseline characteristics, but excluded studies based on cases and matched controls. Only studies with the full text available and peer reviewed in English were included. Full inclusion and exclusion criteria are available in Table 1.

**Table 1:** Inclusion and exclusion criteria for study inclusion eligibility.

Inclusion	Exclusion
Adults aged ≥18 years	Children / animals
Recruited from a general population (populations selected due to age are acceptable provided this is consistent for each ethnic group)	Recruited due to disease or symptom status (e.g. prostate cancer), medication usage, or other ‘non-general’ population (e.g. healthcare workers, pregnant women, marathon runners etc.)
Available PSA test result as raw values, a pooled average (e.g. mean, median, range, centiles) or proportion above or below a certain figure	No available PSA test result
PSA values must be stratified by broad ethnic group	No stratification of PSA level by broad ethnic group
More than one ethnic group reported	Only one ethnic group reported
Observational studies without matching	Matched observational studies
Baseline PSA values from randomised controlled trials	PSA levels reported after intervention in randomised controlled trials
Peer reviewed, full text available in English	Abstracts, full texts not peer-reviewed or available in English

### Search strategy

*PubMed* and *Embase* were searched on the 24^th^ September 2021 and again on the 21^st^ June 2022 to identify studies that reported levels of PSA for at least two ethnic groups. Search terms included prostate-specific antigen *or* PSA *and* ethnicity *or* ethnic group. To capture all ethnicity or ethnic groups, the search included mESH terms for a number of ancestry groups such as African, European, Asian, American Native, and Oceanic, mESH terms for Ethnic Groups and Minority Groups, as well as commonly used terms to describe ethnic groups such as African*, Caucas*, Europ*, Asian*, Indian*, Maori*, Hispanic*, Chinese* etc. The terms White and Black were included if they appeared within three words of ethnic* in attempt to reduce non-specific search returns. Full search terms can be found in Appendix 1. Endnote X9 was used to automatically detect duplicates which was followed by manual detection by one reviewer. Two reviewers independently screened abstracts and full texts for eligibility and conflicts were resolved by discussion with a third reviewer. Cohen’s kappa was calculated to assess interrater reliability.

### Data extraction

Data extraction was completed independently by two reviewers and cross-referenced for discrepancies. The following data were extracted: number and age of patients, country of study, the context in which PSA levels were collected (healthcare records vs PSA test in general population), ascertainment of ethnicity, and PSA measures (median, mean, centiles, proportion above/below) for each ethnic group. Given that age is a factor in PSA variation, age-stratified or age-adjusted levels were extracted where reported.

### Quality assessment

As this systematic review extracted PSA measures from a variety of study designs, an adaptation of the Newcastle-Ottawa scale for cohort studies was used to assess the quality and risk of bias of the included studies, addressing the aims of this review rather than the individual aims of the included papers. The adaptation of the Newcastle-Ottawa assessment can be found in Appendix 2. Two reviewers independently scored each paper based on selection, comparability, and outcome domains for a maximum of nine stars. Conflicts were resolved by discussion. Each study was then classed as ‘good’, ‘fair’, or ‘poor’ based on the Newcastle-Ottawa thresholds.

### Data synthesis

Participants were stratified into the following broad ethnic groups based on the classification used in the included papers: White (including White, Caucasian, European), Black (Black, African American, non-Hispanic Black), Asian (Asian), Hispanic (Hispanic, Latino, Mexican-American), and Other (Other, Maori, Pacific Island, Pacific People). Where studies reported more than one summary statistic for PSA values, the summary statistic most comparable with other extractions was chosen for the final table (most often median and 95^th^ percentile). If statistical significance was not reported, confidence intervals were calculated from the mean and standard deviation where possible to infer significance. Studies that were assessed as poor quality were considered separately to studies that were assessed as fair or good in a narrative sensitivity analysis. Due to significant heterogeneity in the reported measures of PSA, meta-analysis was not possible. Therefore, results were collated and summarised into a narrative synthesis following previously published guidance [20].

## Results

### Database search

**Fig. 1:**
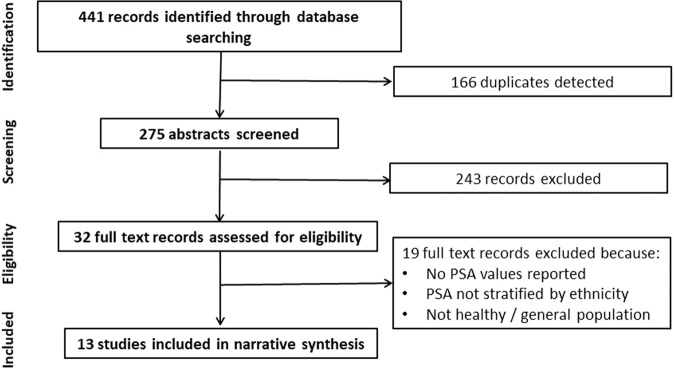
Flow diagram of the study selection process.

The database search returned 441 studies, from which 166 duplicates and 243 irrelevant studies were excluded based on title and abstract. This left 32 studies for full-text review, of which 19 studies were excluded and resulted in 13 studies included in the narrative synthesis (Fig. 1). The level of inter-rater reliability was moderate (0.57, Cohen’s kappa) for abstract screening and substantial (0.64, Cohen’s kappa) for full-text screening.

### Study quality

Just over half (7/13) of the included studies were assessed as high quality (good) using the adapted Newcastle-Ottawa scale, with the remainder rated as poor. The full evaluation of study quality assessment can be found in Table 2.

**Table 2:** Study quality assessment using an adapted version of the Newcastle-Ottawa scale.

Source	Selection				Comparability	Outcome		Rating
	i	ii	iii	iv	v	vi	vii	
Crawford 2011 [17]		*				*	**	Poor
DeAntoni 1996 [18]	*	*	*		**	*	**	Good
Espaldon 2014 [19]	*	*	*		**	*	**	Good
Gray 2003 [29]	*		*	*	**	*	*	Good
Gray 2005 [30]	*		*	*	**	*	*	Good
Lacher 2006 [24]	*	*	*	*	**	*	**	Good
Lacher 2015 [21]	*	*	*		**	*	**	Good
Matti 2021 [28]	*	*	*		**	*		Poor
Rhodes 2012a [22]	*					*	**	Poor
Rhodes 2012b [23]	*					*	**	Poor
Saraiya 2005 [26]	*	*	*		**	*	**	Good
Sarma 2014 [27]	*				**	*	**	Poor
Weinrich 1988 [25]	*	*			**	*		Poor

i: representativeness of cohort, ii: selection of cohorts, iii: assignment of ethnicity, iv: sample size v- comparability of ethnic groups, vi: ascertainment of blood tests, vii: statistical analysis. * 1 star, ** 2 stars.

### Study characteristics

The characteristics of the included studies are summarised in Table 3. A total of 654 197 males aged ≥40 years old from 13 studies were included, although up to 2 313 of these patients (1 808 White and 705 Black men) may have been duplicated through use of the same datasets. By ethnic group, there were 536 201 White (82%), 38 287 Black (6%), 38 232 Asian (6%), 18 029 Pacific Island (3%), 13 614 Maori (2%), 8 885 Hispanic (1%), and 949 Other (<1%) males included. Study countries included the USA and New Zealand.

**Table 3:** Characteristics of the included studies.

Study	Ethnicity, *n*	Age, *years*	Country	Dataset/Cohort	Study quality
		*Mean (SD)*			
Crawford 2011 [17]	14 186 Caucasian 6 367 African-American 949 Other	55.7 (10.3) 54.1 (10.3) 53.4 (9.1)	USA	Health Alliance Plan of Henry Ford Health System	Poor
		*Mean*			
DeAntoni 1996 [18]	70 772 White 4 485 Black 1 543 Latino 900 Asian	61.4 55.5 57.5 59.9	USA	Prostate Cancer Awareness Week	Good
		*Median*			
Espaldon 2014 [19]	296 477 White 25 058 Black 5 749 Latino	70-74 70-74 75-79	USA	Veterans Affairs and Medicare	Good
		*Median*			
Gray 2003 [29]	717 Caucasian 348 Maori 340 Pacific Islander	50–59 40-49 50–59	NZ	Wellington Regional Community Prostate Study	Good
		*Range*			
Gray 2005 [30]	728 Caucasian 353 Maori 344 Pacific Islander	40-69 40-69 40-69	NZ	Wellington Regional Community Prostate Study	Good
		*Median*			
Lacher 2006 [24]	1 476 N-H White 435 N-H Black 485 Mexican-American	60–69 50–59 60–69	USA	NHANES 2001-2004	Good
		*Median*			
Lacher 2015 [21]	1 700 N-H White 560 N-H Black 864 Hispanic	60–69 50–59 50–59	USA	NHANES 2007-2010	Good
		*Median (IQR)*			
Matti 2021 [28]	147 542 European 37 332 Asian 12 913 Maori 17 345 Pacific People	59 (15) 57 (14) 56 (13) 57 (13)	NZ	Northern Cancer Network of NZ	Poor
		*Median*			
Rhodes 2012a [22]	420 White 329 Black	50–59 50–59	USA	OCS & FMHS	Poor
		*Mean (SD)*			
Rhodes 2012b [23]	420 White 328 Black	58.7 (10.4) 57.4 (10.4)	USA	OCS & FMHS	Poor
		*Median*			
Saraiya 2005 [26]	768 N-H White 227 N-H Black 244 Mexican-American	60–69 50–59 50–59	USA	NHANES 2001-2002	Good
		*Median*			
Sarma 2014 [27]	616 Caucasian 150 African-American	50–59 50–59	USA	OCS & FMHS	Poor
		*Median*			
Weinrich 1998 [25]	379 White 348 Black	50–59 50–59	USA	South Carolina Prostate Cancer Programme	Poor

*N-H* Non-Hispanic, *NHANES* National Health and Nutrition Examination Study, *NZ* New Zealand, *OCS & FMHS* Olmsted County Study of Urinary Symptoms and Health Status Among Men & Flint Men’s Health Study, *USA* United States of America.

The PSA values from each study are reported in Table 4, stratified by ethnic group. Nine studies stratified PSA values by age, one study adjusted PSA values for age [21], and three studies did not control for age [17, 22, 23]. PSA levels were summarised by mean (standard deviation (SD)), percentage of men over a certain threshold (1.4 ng/mL and 4.0 ng/mL), median and interquartile range (IQR), 90^th^ percentile, 95^th^ percentile, and age-adjusted mean with standard error (SE). The most common summary statistic used was median with 95^th^ percentile.

**Table 4:** PSA values from included studies, by ethnicity.

		PSA values (ng/ml)						
Study	Age group, *years*	White	Black	Asian	Hispanic	Other		Outcome summary
		*Mean (SD)*	*Mean (SD)*			*Mean (SD)*		
Crawford 2011 [17]	40+	1.02 (0.75)	1.03 (0.77)			0.98 (0.68)		No evidence of differences
		*Mean (SD)*	*Mean (SD)*	*Mean (SD)*	*Mean (SD)*			
DeAntoni 1996 [18]	40–49 50–59 60–69 70–79	0.82 (0.77) 1.2 (1.3) 1.8 (1.9) 2.3 (2.3)	0.87 (0.92) 1.4 (1.6) 2.0 (2.4) 2.5 (2.7)	0.85 (0.57) 1.3 (1.6) 1.8 (1.9) 2.3 (2.3)	0.73 (0.71) 1.3 (1.6) 1.8 (2.1) 2.0 (2.3)			White < Black * (age groups 50–59 and 60–69)
		*PSA > 4.0 (%)*	*PSA > 4.0 (%)*		*PSA>4.0 (%)*			
Espaldon 2014 [19]	65–69 70–74 75–79 80–84 ≥85	5.6 7.0 9.9 13.1 17.0	9.8 13.1 17.8 24.0 27.4		5.1 9.0 10.6 13.6 18.3			White/Latino <Black *** (overall, not age-stratified)
		*Median*, 95th				*Median*, 95th		*Median*, 95th
Gray 2003 [29]	40–49 50–59 60–69	0.7, 2.1 0.9, 2.8 1.4, 4.0				Maori 0.6, 1.6 0.8, 2.3 1.5, 4.6	Pacific 0.7, 1.5 0.8, 3.0 1.1, 3.0	White > Maori* (age-adjusted mean)
		*PSA > 4.0 (%)*				*PSA > 4.0 (%)*	*PSA > 4.0 (%)*	
Gray 2005 [30]	40–49 50–59 60–69	0.4 3.5 11.0				Maori 0.6 3.2 13.2	Pacific 1.3 3.7 12.7	No evidence of differences
		*Median*, 95th	*Median*, 95th		*Median, 95*^th^			
Lacher 2006 [29]	40–49 50–59 60–69 ≥70	0.70, 2.37 0.90, 3.11 1.10, 4.98 1.70, 8.66	0.70, 2.00 0.80, - 1.50, 10.30 -		0.71, 1.87 0.90, 3.16 1.00, - 1.60, -			White < Black* (95 percentile, age 60–69)
		*Mean (SE)*^ǂ^	*Mean (SE)*^ǂ^		*Mean (SE)*^ǂ^			
Lacher 2015 [21]	40+	1.03 (0.02)	1.25 (0.07)		1.11 (0.06)			White < Black*
		*95th*		*95th*		*95th*	*95th*	
Matti 2021 [28]	40–44 45–49 50–54 55–59 60–64 65–69 70–74 75–79	1.60 1.90 2.41 3.19 4.01 4.90 5.87 7.39		1.60 1.80 2.29 3.10 3.90 4.81 5.87 7.17		Maori 1.60 1.90 2.51 3.39 4.48 5.58 7.10 9.39	Pacific 1.51 1.90 2.61 3.29 4.48 5.99 7.77 10.7	No evidence of differences
		*PSA>1.4, n (%)*	*PSA>1.4, n (%)*					
Rhodes 2012a [22]	N/A	146 (34.8)	109 (33.1)					No evidence of differences
		*Median (IQR)*	*Median (IQR)*					
Rhodes 2012b [23]	N/A	1.2 (0.8–1.8)	1.0 (0.5–1.7)					White > Black*** (unadjusted for age)
		*Median*, 90th	*Median*, 90th		*Median, 90*^*th*^			
Saraiya 2005 [26]	40–49 50–59 60–69 70+	0.73, 1.53 0.82, 2.42 0.99, 3.25 2.02, 6.30	0.65, 1.51 0.90, 3.25 1.59, 5.33 1.84, -		0.78, 1.55 0.83, - 1.12, 3.19 1.40, -			No evidence of differences
		*Median*, 95th	*Median*, 95th					
Sarma 2014 [27]	40–49 50–59 60–69 70+	0.7, 1.8 0.9, 2.9 1.4, 4.6 2.1, 7.1	0.8, 2.4 0.8, 3.3 1.1, 6.1 1.4, 5.6					No evidence of differences
		*Median*, 95th	*Median*, 95th					
Weinrich 1998 [25]	50 – 59 60 – 69	0.8, 2.7 1.0, 4.9	0.9, 3.8 1.1, 5.7					White < Black ** in 50–59 age group (age-stratified mean)

Where more data were available than the data presented in the table, summary statistics that were most comparable to other entries were selected and recorded.

- values suppressed due to large standard error or small sample size, ^ǂ^age-adjusted summary statistic, 90^th^ 90^th^ percentile, 95^th^ 95^th^ percentile, *p < 0.05, **p < 0.01, ***p < 0.001.

### Narrative synthesis

Ten studies reported PSA values for White and Black men, all conducted in the USA. Half of these studies found higher PSA levels in Black men compared with White men [18, 19, 21, 24, 25], one study found the opposite [23], while the remaining four studies found no difference between men of both groups [17, 22, 26, 27]. Of the five studies that did not find any evidence of differences, or found that White men had higher PSA levels than Black men, four of these studies were rated as poor quality [17, 22, 23, 27]. Incidentally, three of these studies did not stratify by age [17, 22, 23] and the mean age of White men was greater than Black men in two of these [17, 23].

All of the five studies that compared PSA levels of Hispanic men to White and Black men were assessed as good quality [18, 19, 21, 24, 26]. One of these studies found fewer Hispanic men experienced PSA values over 4 ng/mL compared to Black men, and while this was not an age-adjusted or age-stratified calculation, Hispanic men had a higher median age range than Black men [19]. The remaining four studies had a much smaller sample size of Hispanic men and found no evidence of differences in PSA values between Hispanic and Black men. None of the five studies reported a difference in PSA levels between Hispanic and White men; indeed, the summary values reported for these men appeared consistently similar across each study.

Only two studies reported PSA levels for Asian men, and both found no difference between Asian men and men of other ethnic groups [18, 28].

Finally, there was no evidence of differences in PSA levels between the Maori and Pacific Island ethnic groups in any of the three studies that reported PSA levels in New Zealand [28–30]. A difference was found in the age-adjusted mean difference between Maori and White men, with significantly higher PSA levels reported for White men [29]. However, in an almost identical cohort, this difference was not observed in Grey, et al. (2005) when assessing proportions of men with a PSA level above 4 ng/mL [30].

## Discussion

### Key findings

This systematic review found evidence that Black males had higher PSA levels than White males in the USA, with no published evidence from other countries with significant Black populations. Multiple studies suggested Hispanic men have similar PSA levels to White men in the USA, with one study reporting lower PSA levels in Hispanic men compared to Black men. In New Zealand, Maori men were found to have lower levels of PSA than White men and there were no differences between Maori men and Pacific Island men. There was no evidence of differences between the PSA levels of Asian men and men of other ethnic groups.

### Strengths and limitations

The overarching strength of this study was the inclusion of 654 197 men. Importantly, studies were only selected if PSA values were collected for men within the same country to control for differences in healthcare systems and inter-country cultures, and age-stratified or age-adjusted PSA values were meticulously extracted where possible to control for the effects of age.

A weakness was the availability of studies limited to the USA and New Zealand and a considerable underrepresentation of the Asian and Mixed ethnicities, major ethnic groups in many countries. A meta-analysis would have provided further certainty of differences in PSA values across the ethnic groups, although this was not possible due to the heterogeneity of reported PSA measures. Publication bias may have resulted in a higher number of publications reporting ethnic differences in PSA values. However, the outcome of four of the included studies was prostate cancer risk or urological outcomes [17, 22, 23, 29], thus any reported ethnic differences in PSA values from the patient characteristics of these studies should have been free from publication bias. Indeed, Rhodes, *et al*. (2012b) [23] reported higher PSA levels in Black men compared to White men in their patient characteristics.

### Comparison to existing literature

Ethnicity was found to be a source of significant heterogeneity in a recent systematic review assessing age-adjusted reference ranges in apparently healthy men: controlling for ethnicity in 10-year age intervals reduced study heterogeneity by 13% from 99% (*I*^2^ statistic) [16]. The remaining heterogeneity may have been explained, in part, by differences in inter-country cultures and healthcare systems, which was not controlled for. The study concluded ethnicity was an important parameter that influenced PSA levels and should be considered when clinically assessing PSA values.

In a prostate cancer cohort in the UK including Black and White men, Black men were found to have higher PSA levels than White men at the point of diagnosis [31]. Despite this, Black men were diagnosed at similar clinical stages and had similar Gleason scores, and interestingly, preliminary data from the authors suggested there was no difference in prostate cancer mortality between Black and White men [32]. This starkly contrasts with abundant data from the USA reporting poorer prognosis in Black men [33] which may be attributed to the differences in healthcare systems between the two countries: access to healthcare in the USA varies significantly by income and deprivation, whereas healthcare in the UK is universally free at point of access. As deprivation levels are higher in Black men in the USA compared to White men, Black men may be less able or likely to access healthcare [34]. Asian men in the UK were reported to have lower incidence of prostate cancer than White men, with lower PSA levels at diagnosis and less aggressive disease at presentation [35].

### Clinical implications

The findings of this systematic review shed some light on PSA levels of men across different ethnic groups. Further research is needed to determine whether these differences are enough to warrant introducing ethnicity into guidance for interpreting PSA levels, and what the implications of that may be. The accuracy of PSA for the diagnosis of prostate cancer in different ethnic groups is unknown, as is whether higher PSA levels observed in Black men are due to higher prevalence of prostate cancer in that group, or whether higher PSA levels in Black men could be contributing to overdiagnosis. Any amendments to current guidance for interpreting PSA based on ethnicity will need to be carefully assessed with thorough modelling and evaluation taking into account prostate cancer incidence, stage at diagnosis and mortality to ensure it was reducing, rather than increasing, health inequalities in prostate cancer diagnosis.

## Conclusion

Healthy Black men have higher PSA levels than White or Hispanic men, which reflects the higher rates of prostate cancer diagnosis in Black men. It is not known whether Black men are diagnosed with similar rates of clinically significant prostate cancer to White men, or whether raised PSA values are contributing to overdiagnosis in Black men. Future research needs to consider the impacts of PSA thresholds in Black men for triggering prostate cancer investigation, and whether ethnicity specific PSA thresholds could help to reduce the ethnic inequalities in prostate cancer diagnosis.

## Supplementary information

Full search terms

Adaptation of the Newcastle-Ottawa quality assessment scale

## Data Availability

Template data extraction forms, data extracted from included studies, and data used for analysis can be supplied from the corresponding author upon request.
