# Supplementary material for: Ethnic differences in prostate-specific antigen levels in men without prostate cancer: a systematic review
Source: Prostate Cancer Prostatic Dis. 2022 Dec 1;26(2):249–56. doi: 10.1038/s41391-022-00613-7 (PMC10247367; doi:10.1038/s41391-022-00613-7)
Supplement: Supplementary file 1 — Full search terms [file 41391_2022_613_MOESM1_ESM.docx]

Full search terms

Used to search Medline and EMBASE on 24^th^ September 2021 and 21^st^ June 2022.

| 1 | exp Prostate-Specific Antigen/ |
| --- | --- |
| 2 | psa*.ti,ab. |
| 3 | prostate specific antigen*.ti,ab. |
| 4 | 1 or 2 or 3 |
| 5 | exp African Continental Ancestry Group/ |
| 6 | African*.ti,ab. |
| 7 | Negro*.ti,ab. |
| 8 | Afro*.ti,ab. |
| 9 | (Black adj3 ethnic*).ti,ab. |
| 10 | (Black adj3 race*).ti,ab. |
| 11 | (Black adj3 racial*).ti,ab. |
| 12 | 5 or 6 or 7 or 8 or 9 or 10 or 11 |
| 13 | exp European Continental Ancestry Group/ |
| 14 | Caucas*.ti,ab. |
| 15 | Europ*.ti,ab. |
| 16 | (White adj3 ethnic*).ti,ab. |
| 17 | (White adj3 race*).ti,ab. |
| 18 | (White adj3 racial*).ti,ab. |
| 19 | 13 or 14 or 15 or 16 or 17 or 18 |
| 20 | exp Asian Continental Ancestry Group/ |
| 21 | Asian*.ti,ab. |
| 22 | Indian*.ti,ab. |
| 23 | Pakistani*.ti,ab. |
| 24 | Bangladeshi*.ti,ab. |
| 25 | Bengalis*.ti,ab. |
| 26 | Kashmiris*.ti,ab. |
| 27 | Gujaratis*.ti,ab. |
| 28 | Tamils*.ti,ab. |
| 29 | Sri Lankan*.ti,ab. |
| 30 | Chinese*.ti,ab. |
| 31 | Japanese*.ti,ab. |
| 32 | Oriental*.ti,ab. |
| 33 | Thai*.ti,ab. |
| 34 | Phillipino*.ti,ab. |
| 35 | Filipino*.ti,ab. |
| 36 | Taiwanese*.ti,ab. |
| 37 | 20 or 21 or 22 or 23 or 24 or 25 or 26 or 27 or 28 or 29 or 30 or 31 or 32 or 33 or 34 or 35 or 36 |
| 38 | exp American Native Continental Ancestry Group/ |
| 39 | Native American*.ti,ab. |
| 40 | Native Canadian*.ti,ab. |
| 41 | Native Alaskan*.ti,ab. |
| 42 | American Native*.ti,ab. |
| 43 | Canadian Native*.ti,ab. |
| 44 | Alaskan Native*.ti,ab. |
| 45 | 38 or 39 or 40 or 41 or 42 or 43 or 44 |
| 46 | exp Oceanic Ancestry Group/ |
| 47 | Aborigin*.ti,ab. |
| 48 | Indigenous p*.ti,ab. |
| 49 | Maori*.ti,ab. |
| 50 | Pacific Island*.ti,ab. |
| 51 | 46 or 47 or 48 or 49 or 50 |
| 52 | Inuit*.ti,ab. |
| 53 | Eskimo*.ti,ab. |
| 54 | Aleut*.ti,ab. |
| 55 | 52 or 53 or 54 |
| 56 | Arab*.ti,ab. |
| 57 | Bedouin*.ti,ab. |
| 58 | Semit*.ti,ab. |
| 59 | Jew*.ti,ab. |
| 60 | Israeli*.ti,ab. |
| 61 | 56 or 57 or 58 or 59 or 60 |
| 62 | Hispanic*.ti,ab. |
| 63 | Latino*.ti,ab. |
| 64 | 62 or 63 |
| 65 | 12 and 19 |
| 66 | 12 and 37 |
| 67 | 12 and 45 |
| 68 | 12 and 51 |
| 69 | 12 and 55 |
| 70 | 12 and 61 |
| 71 | 12 and 64 |
| 72 | 37 and 45 |
| 73 | 37 and 51 |
| 74 | 37 and 55 |
| 75 | 37 and 61 |
| 76 | 37 and 64 |
| 77 | 45 and 51 |
| 78 | 45 and 55 |
| 79 | 45 and 61 |
| 80 | 45 and 64 |
| 81 | 51 and 55 |
| 82 | 51 and 61 |
| 83 | 51 and 64 |
| 84 | 55 and 61 |
| 85 | 55 and 64 |
| 86 | 61 and 64 |
| 87 | 65 or 66 or 67 or 68 or 69 or 70 or 71 or 72 or 73 or 74 or 75 or 76 or 77 or 78 or 79 or 80 or 81 or 82 or 83 or 84 or 85 or 86 |
| 88 | exp Ethnic Groups/ |
| 89 | exp Minority Groups/ |
| 90 | ethnic*.ti,ab. |
| 91 | race*.ti,ab. |
| 92 | racial*.ti,ab. |
| 93 | 90 or 91 or 92 |
| 94 | inter*.ti,ab. and 93 |
| 95 | intra*.ti,ab. and 93 |
| 96 | difference*.ti,ab. and 93 |
| 97 | 88 or 89 or 94 or 95 or 96 |
| 98 | 87 or 97 |
| 99 | exp Reference Values/ |
| 100 | exp Reference Standards/ |
| 101 | reference value*.ti,ab. |
| 102 | reference range*.ti,ab. |
| 103 | reference interval*.ti,ab. |
| 104 | reference standard*.ti,ab. |
| 105 | distribution*.ti,ab. |
| 106 | 99 or 100 or 101 or 102 or 103 or 104 or 105 |
| 107 | 4 and 98 and 106 |
