# Supplementary material for: Ethnic differences in prostate-specific antigen levels in men without prostate cancer: a systematic review
Source: Prostate Cancer Prostatic Dis. 2022 Dec 1;26(2):249–56. doi: 10.1038/s41391-022-00613-7 (PMC10247367; doi:10.1038/s41391-022-00613-7)
Supplement: Supplementary file 2 — Adaptation of the Newcastle-Ottawa quality assessment scale [file 41391_2022_613_MOESM2_ESM.docx]

ADAPTATION OF THE NEWCASTLE - OTTAWA QUALITY ASSESSMENT SCALE

*A study can be awarded a maximum of one star for each numbered item, other than Outcome 2 and Comparability, in which a maximum of two stars can be given*.

**Selection**

1. Representativeness of the cohort
   1. Truly representative of a general / healthy population *
   2. Somewhat representative of a general / healthy *
   3. Selected group of participants e.g. hospital workers, marathon runners etc.
   4. No description of the derivation of the cohort
2. Selection of ethnic groups
   1. Drawn from the same community *
   2. Drawn from a different source
   3. No description of the derivation of the ethnic groups
3. Assignment of ethnicity
   1. Self-reported *
   2. Secure record (e.g. medical records) if evidence the ethnicity came from a self-report *
   3. Determined by clinician or researcher
   4. No description
4. Sample size:
   1. Calculated and described *
   2. Not calculated or described

**Comparability**

1. Comparability of ethnic groups
   1. Controls for most important factor (e.g. age) **
   2. Not controlled for any factors

**Outcome**

1. Ascertainment of blood test
   1. Secure records (e.g. medical records, laboratory results) *
   2. Self-report
   3. No description
2. Statistical test to compare differences in blood test values across ethnic groups
   1. Clearly described and appropriate, and the measurement of the association is presented with the probability level (*p* value), confidence intervals (CIs), or mean (SD) to calculate CIs **
   2. Statistical test used and p-value, CIs, or mean (SD) provided, but absent or unclear description of statistical test used *
   3. The statistical test is not appropriate, not described, or incomplete.

**Thresholds for converting the Newcastle-Ottawa scales to AHRQ standards (good, fair, and poor):**

**Good quality:** 3 or 4 stars in selection domain *AND* 1 or 2 stars in comparability domain *AND* 2 or 3 stars in outcome/exposure domain

**Fair quality:** 2 stars in selection domain *AND* 1 or 2 stars in comparability domain *AND* 2 or 3 stars in outcome/exposure domain

**Poor quality:** 0 or 1 star in selection domain *OR* 0 stars in comparability domain *OR* 0 or 1 stars in outcome/exposure domain
